# Knowledge, Attitudes and Perceptions towards COVID-19 Vaccinations: A Cross-Sectional Survey in Pakistan

**DOI:** 10.3390/medicina59020272

**Published:** 2023-01-31

**Authors:** Asima Bibi, Sameen Abbas, Saima Mushtaq, Atika Mansoor, Ivan R. Green, Tauqeer Hussain Mallhi, Yusra Habib Khan, Amjad Khan

**Affiliations:** 1 Department of Pharmacy, Quaid-i-Azam University, Islamabad 45320, Pakistan; 2 Department of Healthcare Biotechnology, Atta-ur-Rahman School of Applied Biosciences, National University of Sciences and Technology, Islamabad 44000, Pakistan; 3 Institute of Biomedical and Genetic Engineering (IBGE), KRL Hospital, Islamabad 44000, Pakistan; 4 Department of Chemistry and Polymer Science, University of Stellenbosch, Matieland, Stellenbosch 7600, South Africa; 5 Department of Clinical Pharmacy, College of Pharmacy, Jouf University, Sakaka 72388, Saudi Arabia

**Keywords:** COVID-19, vaccine, knowledge, attitude, perception, survey, questionnaire

## Abstract

*Background and Objectives:* Several vaccines have been approved for the prevention of the coronavirus disease, discovered on 31 December in Wuhan, China. Pakistan procured vaccines from various countries. However, the lack of knowledge and reluctance of the general population to embrace the use of the vaccines are considered to be the major determinant of the slow vaccination rate. Hence, it is necessary to evaluate the willingness of the general population about their perception of the COVID-19 vaccination. *Materials and Methods:* A cross sectional survey based on a self-structured questionnaire comprising 18 questions was conducted (from 21 April–21 June) on 400 Pakistani participants to evaluate their knowledge, attitude, and perception towards the COVID-19 vaccination. Chi-square independent t-test and one-way Anova including a multiple step wise linear regression were used to draw conclusions about the results. *p* < 0.05 was considered significant. *Results:* A total of 400 participants responded in the knowledge, attitude, and perception (KAP) survey of which 46.5% were female and 53.5% were male. The mean age of participants was 36.08 years. This survey showed a poor knowledge (50.5%), a fair attitude (75.1%) and a poor perception (58.1%) towards the COVID-19 vaccination. Higher mean knowledge and attitude scores were reported in the age group 21–40, females, and unmarried urban citizens. Regression analysis showed that age, education, residence, and employment status influenced the knowledge and perception score to a considerable extent. *Conclusions:* The findings reflect an inadequate knowledge and perception on the one hand, but a better attitude towards the COVID-19 vaccination. This knowledge attitude and perception (KAP) survey will help in better understanding the opinion of the general population towards vaccination, and will be useful for policy makers and health care authorities aiming to increase the vaccination rate.

## 1. Background

After the Severe Acute Respiratory Syndrome (SARS) and Middle East Respiratory Syndrome (MERS) outbreak, a new virus was discovered on 31 December in Wuhan, China, named 2019 n-CoV by the World Health Organization (WHO), and later named SARS-CoV-2 by the International Committee on the taxonomy of viruses [1]. The WHO declared COVID-19 to be the sixth Public Health Emergency of International Concern (PHEIC), and later declared it to be a global pandemic on 11 March 2020 (WHO, 2005). The first COVID-19 case was reported in Pakistan on 26 February 2020 [2]. Since then, the number of cases increased rapidly.

Persons infected with COVID-19 develop the common conditions of fever, cough, shortness of breath, sore throat, nasal congestion, weakness, fatigue, and dyspnea [3,4,5]. Various risk factors are involved with the complexity of COVID-19. These risk factors are: old age, respiration distress, and various chronic co-morbid conditions [6,7] Other risk factors includes respiratory distress, sepsis, metabolic acidosis, arrhythmia heart failure, kidney failure, and hypoxic encephalopathy [8].

Treatment strategies were initially mainly focused on corticosteroids, blood thinners, and neutralizing antibodies, due to the lack of a specific cure, such as vaccination [9]. After an extensive development phase, vaccination became one of the best treatment strategies recommended by the WHO to generate herd immunity in the general population [10,11]. Despite supply challenges, the Pakistan government acquired 40 million COVID-19 vaccines from China, the WHO, USA, UK and Germany, in order to be able to vaccinate an estimated 70 million people [12,13,14,15,16]. Statistics shared by the Government of Pakistan showed that only 18.2% of the Pakistan population is fully vaccinated [17]. Pakistan has a history of a relatively low vaccination rate for a variety of vaccine preventable diseases, such as HBV and polio [18]. Unless this COVID-19 low vaccination rate is not seriously addressed, it will take an unnecessarily longer time for the general population to return a semblance of normalcy from this pandemic [19].

It is well known that, generally, any new medical intervention has its own acceptability rate among the general population, and thus the acceptance of the COVID-19 vaccine, along with its distribution and proper utilization to every member of society, is also very important. [20]. Previous reported data showed that those reluctant to take the COVID-19 vaccination due to safety concerns totaled, in the USA, 33% of general participants and 50% of health care workers [21]; in Turkey, 45.3% of general participants and 42.2% of health care workers; and in Oman, 23% of general respondents and 40% of healthcare workers [22,23,24,25,26]. In the USA, its acceptance was 50%, in France its acceptance was only 62% [20,27]. While in Italy, acceptance of the COVID-19 vaccination was 59% [28,29]. Similarly, one of China’s surveys about COVID-19 vaccination declared that only one half, that is 54% pf the population, was willing to have vaccination [30]. Several important factors, such as health knowledge, serve as being important for the participants in increasing their acceptance, as more knowledge by the general population towards breakouts such as the COVID-19 pandemic, its vaccination and potential benefits, coupled with its precautionary measures, contributes in a better implementation of health system facilities [30,31]. Similarly, attitude and perception are the two primary cognitive factors that play a vital role in the vaccination coverage rate of COVID-19.

Knowledge, attitude, and perception (KAP) surveys mostly help to identify knowledge gaps and behavior patterns of the general population on the basis of their socio-demographics, in order to implement effective public health interventions [32]. This study aimed to determine the knowledge, acceptance, and perception of the COVID-19 vaccine among the Pakistani population.

## 2. Methodology

### 2.1. Study Design

Cross sectional studies were performed to assess knowledge, attitude, and perception of the Pakistani population towards the COVID-19 vaccination.

### 2.2. Study Setting

The study was conducted on the general population of all the provinces (Punjab, Sindh, Khyber Pakhtunkhwa, Balochistan) of Pakistan.

### 2.3. Study Duration

The study was carried out from April–June 2021 through an online questionnaire, which was distributed on different social media platforms (e.g., Facebook and WhatsApp). During this time duration of April to June (3rd and 4th wave of COVID-19), a community-based national survey was not possible. So, relying on online social media links, the questionnaire was posted/reposted to local people living in different areas of Pakistan. In this online survey, answers to all questions was mandatory for final submission. 

### 2.4. Inclusion and Exclusion Criteria

Participants were 18 years or older and Pakistani residents, having an easy access to the Internet and were voluntary participants. People below the targeted age of 18 years were excluded from this study.

### 2.5. Sample Size and Sampling Technique 

Initially, the convenient sampling technique was used for sample size estimation. In this survey, thousands of participants could be included, however, due to the limited time period, sample size was calculated from the estimated current population of Pakistan by using the Rao-soft calculator to have an idea of the least number of participants that must be included in this survey. The current population of Pakistan is 213,222,917 as per 2017 Census of Pakistan. With a 95% confidence interval, 50% population representation and 5% margin of error, a 385 sample size was calculated by using the Rao-soft calculator. However, data from 400 participants was collected. It was a limited sample size because of the limited time duration of survey during the 3rd and 4th wave.

### 2.6. Study Tool

A self-structured 18-item questionnaire, along with the appropriate demographics, was prepared and divided into three sections. In addition to demographics, six questions explored knowledge about COVID-19 while eight questions focused on attitude and four questions focused on perception of participants towards the COVID-19 vaccination. 

### 2.7. Questionnaires Development and Validation

A self-structured questionnaire was designed based on a previous literature review. After an extensive literature review, the questionnaire was designed in English [33]. The English version of the questionnaire was translated into Urdu by using a back-to-back translation procedure [34]. This questionnaire was tested for its reliability and internal consistency. The internal consistency of the knowledge, attitude, and perception (KAP) survey questionnaire calculated by Cronbach’s alpha was 0.720 for knowledge, 0.642 for attitude, and 0.629 for perception, and found to be in an acceptable range. An initial pilot study was performed among 20 participants to evaluate its acceptability and consistency, but these results were not included in the final study.

### 2.8. Scoring Criteria and Statistical Analysis

The scoring criteria was based on the original bloom’s cut-off point used in previous studies conducted on dengue fever anticipation in male people of the Maldives and Bangkok in 2007, as well as a KAP study performed on COVID-19 among Chronic Disease Patients in Northwest Ethiopia in 2020 [35,36]. Criteria of bloom’s cut-off point were 80–100% (good), 60.0–79.0% (fair), and ≤59.0% (poor). In statistical analysis categorical variables were represented in form percentages and frequencies and Chi Square Independent was used to analyze significant association between demographics and knowledge, attitude, and perception. Independent t-test (for two groups) and one-way ANOVA (for more than two groups) were used to measure association within groups. Similarly, multiple linear regression model was used to analyze the impact of an independent variable over a dependent variable. The statistical software package for social sciences (IBM SPSS for Windows, Version 21.0. SPSS Inc., Chicago, IL, USA) was used to evaluate the data. *p*-value less than 0.05 are considered significant.

### 2.9. Ethical Approval

This survey was conducted after ethical approval from the institutional research and ethics forum of Rawalpindi Medical University (Vide letter number: 64/IREF/RMU/2021, Dated 23 April 2021). Respondents were clearly informed about the purpose of the study and privacy of their data was also assured.

## 3. Results

### 3.1. Demographics of Knowledge, Attitude and Perception Study

Age, gender, marital status, employment, education status, and residence of participants are articulated in Table 1. All these were categorical variables to facilitate statistical analysis in the form of frequencies and percentages to be performed. In all, 400 participants completed the survey. Both males 53.5% (n = 214) and females 46.5% (n = 186) participated in the study. The mean age of participants was 36.08 years. (S.D 15.54). The majority of the respondents were from age group 21–40 years. Most of the participants were urban 281 (70.2%) and married citizens 220 (55.0%). In the educational category, 43.22% (n = 172) of the participants had a higher education or below, while those with a graduate level of education were 30.3% (n = 121). Participants having a postgraduate level of education were 26.7% (n = 107). Participants having government jobs account for 20.3% (n = 81), non-government employees were 18.9% (n = 75), unemployed 16.9% (n = 67), retired 9.1% (n = 36), self-employed 8.0% (n = 32) and students 27.3% (n = 109). This classification illustrates a greater number of government employee participation in the study.

### 3.2. Frequency of Response to Knowledge

**Figure 1:**
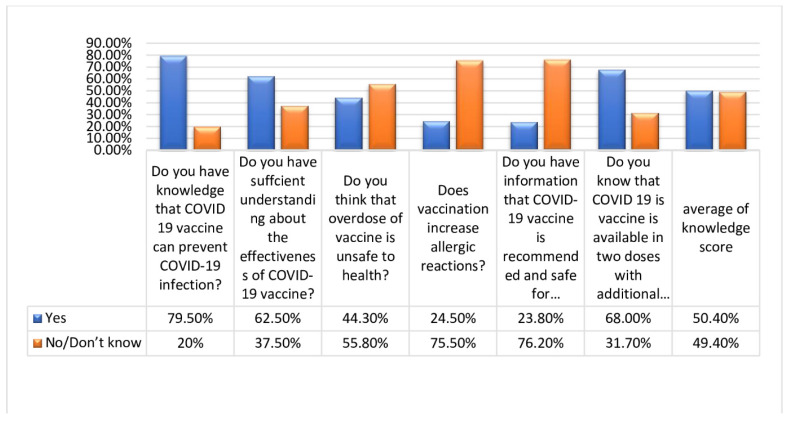
Response to knowledge of the vaccination.

Knowledge was evaluated by six questions about several aspects of the COVID-19 vaccination, with a corresponding scale ranging from 0–6. Responses were scored as 0 for no/don’t know and 1 for yes. Total score was calculated by the sum of six knowledge scores and ranged from 0–6. This led to the finding that 318 (79.5%) know about the COVID-19 vaccination, 250 (62.5%) know about the effectiveness of the vaccination, 177 (44.3%) responded that it is unsafe to use an overdose of vaccination, 302 (75.5%) responded that vaccination cannot cause allergic reactions, while 305 (76.2%) don’t know that the vaccine is recommended for pregnant women and 272 (68.0%) of the participants reported that vaccination is available in two doses with an additional booster dose becoming available at a later stage. Figure 1 also shows the response to knowledge.

### 3.3. Frequency of Response to Attitude

**Figure 2:**
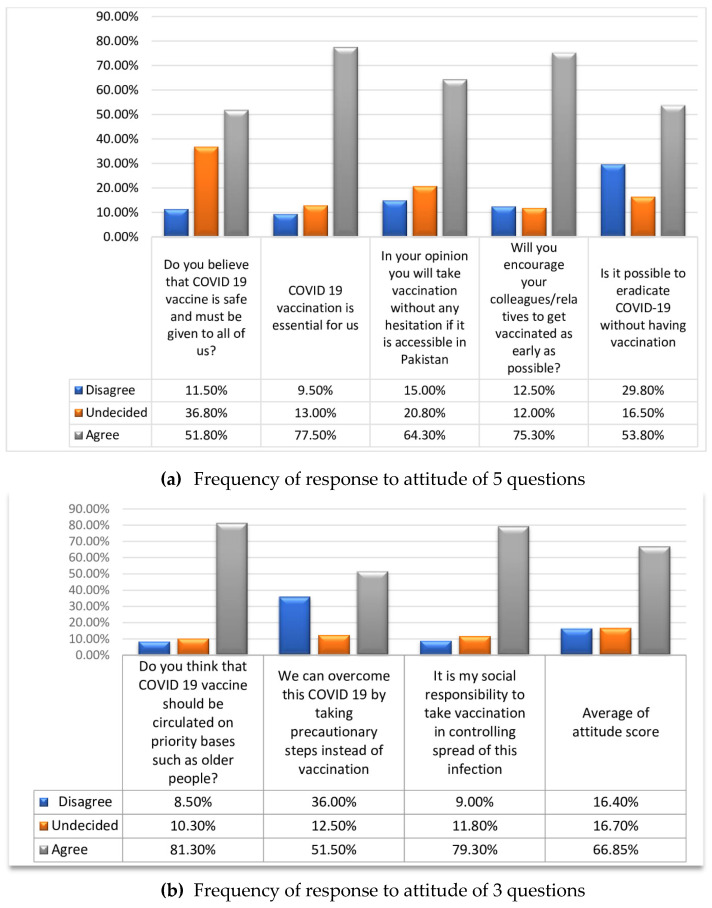
Response to attitude of the vaccination.

Attitude was evaluated by eight questions. Each question was scored as disagree, undecided and agree and scaled as 0, 1 and 2 respectively. Scoring scale ranged from 0–16. In these eight questions of attitude assessment, 400 participants responded, out of which: 207 (51.8%) agreed that the vaccine is safe; 310 (77.5%) agreed that the vaccine is essential; 257 (64.3%) agreed that they will take the vaccination when it becomes available in Pakistan; 301 (75.3%) responded that they will encourage their friends, family, and relatives to get vaccinated; 215 (53.8%) agreed to the response that COVID-19 eradication without vaccination is impossible; 325 (81.3%) agreed that the vaccine should be circulated on a priority basis; 206 (51.5%) believed that by taking precautionary measures instead of vaccination COVID-19 could be eradicated; and 317 (79.3%) agreed that vaccination is their social responsibility to control the spread of COVID-19. Similarly, Figure 2 also shows response to attitude.

### 3.4. Frequency of Response to Perception

**Figure 3:**
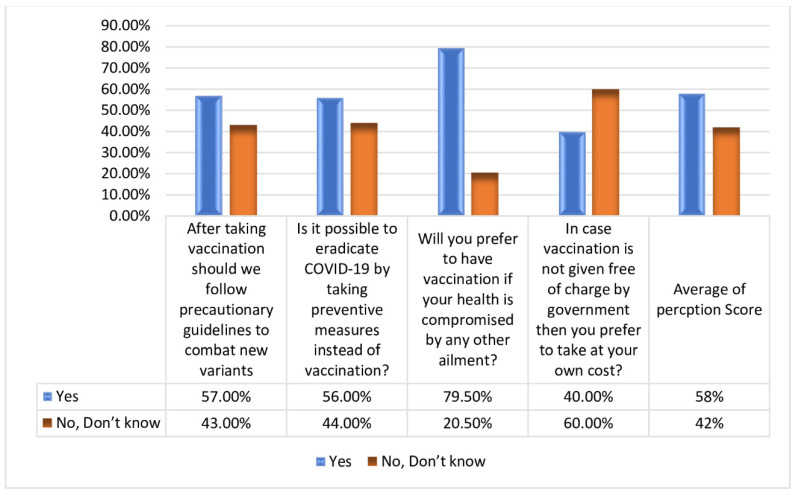
Response to perception of the vaccination.

Perception was assessed by four questions, which were scored as yes, no, do not know, and scaled as 0, for no/do not know and 1 was for a yes response. Scoring scale ranged from 0–4. Responses of participants showed that: 57.0% (n = 228) indicated that after taking the vaccination they should follow guidelines to combat new variants; 56.0% (n = 224) believe that COVID-19 can be eradicated by taking preventive measures instead of vaccination; 79.5% (n = 318) indicated that they prefer to have the vaccination even if their health is compromised by any other ailment; and 60.0% (n = 240) responded that they could not afford vaccination at their own cost if it was not given free of charge by the state. Figure 3 shows response to perception of the participants towards vaccination.

### 3.5. Categorization of Participant’s Score and Their Association with Demographics

Characterization of the participants’ response based on blooms’ cut-off points. Criteria of bloom’s cut-off point were 80–100% (good), 60.0–79.0% (fair), and ≤59.0% (poor). Similarly for knowledge, this score was determined from scale 0–6, for attitude score, 0–16 and for perception score, 0–4. Responses regarding knowledge, age group of participants and the maximum number of ‘fair knowledge’ was observed in people of the age group of 21–40 years. In the gender category, both male and female participants had an equal level of ‘fair knowledge’. In the marital status group, married people had a high rate of fair knowledge. Students and government employees also had a high rate of fair knowledge. Chi-square analysis was used to find any significant association between demographics and knowledge related questions about COVID-19 vaccination. A significant relationship was found between education, employment, and the residence group. 

In responses regarding attitude, participants in the age group of 21–40 years showed a good attitude response. In the gender category, female participants had a high level of ‘good attitude score’. In the marital status group, unmarried people had a high rate of good attitude. Students and urban citizens also had a high rate of good attitude. A significant relationship was found between age, education, marital status, employment, and the residence group. 

In responses about perception, participants in the age group of 21–40 years showed a fair attitude response. In the gender category, male participants had a high level of ‘fair perception’. In the marital status group, married people had a high rate of fair perception. Students and urban citizens also had a high rate of fair perception. No significant association of perception score was noticed with any demographics’ variable. Table 2 summarizes the *p* value obtained.

### 3.6. Analysis of Mean Knowledge, Attitude and Perception

To estimate association within groups with mean knowledge, an independent *t*-test (for two groups) including one for more than two groups and a one-way ANOVA test was performed. The mean count of knowledge was considerably higher among participants aged 21–40 years. Females, unmarried, graduates or below, students, and urban citizens had a higher mean knowledge score. It was found that the mean knowledge score is significantly associated with age, gender, marital status, residence, and employment status.

The mean score of attitude was appreciably higher among participants in the age group of 21–40 years. In terms of gender, females had a high mean attitude score. The educational level of participants also plays a role since a higher mean score of attitude was found in postgraduates and similarly in unmarried and non-government participants. Urban residents also showed a higher mean score. Mean attitude is significantly related with age, gender, education level, marital status, residence, and employment status. 

The mean score of perception was considerably higher among participants aged 21–40 years. In terms of gender, females have a high mean perception score. Educational level is a factor contributing to a higher mean score of perception in graduates, while unmarried and student participants also showed a higher mean perception score, as did urban residents. In this survey, the mean attitude is significantly linked with age, gender, education level, marital status, residence, and employment status. Table 3 below shows association within groups with mean knowledge, mean attitude and mean perception score.

### 3.7. Factors Affecting Knowledge, Attitude and Perception Response on the Use of Vaccine

A multiple linear regression model was used to analyze the impact of an independent variable over a dependent variable, as illustrated in Table 4. Age, education, residence, and employment status influenced the knowledge score to a considerable extent. Gender and marital status had no significant impact on the knowledge score. Correlation analyses shows that the relationship between the dependent and independent variable is a reliable factor for further analysis. Perception score was significantly influenced by age, education, and residence.

## 4. Discussion

In order to overcome the aftermath of the COVID-19 pandemic, the implementation of COVID-19 vaccination is the best if not the ideal solution. After an extensive development phase and positive responses of clinical trials, various countries approved specific vaccines for further implementation. Although various campaigns have been implemented to increase knowledge about vaccination and previous studies also suggests that COVID-19 vaccines are safe and effective in general, based on the billions of doses administered worldwide and the rare incidence of adverse events only in at-risk group [37]. However, due to the newness of this disease, it poses a serious question for policy-makers regarding the knowledge, attitude, and perceptions of the general population about receiving the COVID-19 vaccination. The present survey has been conducted to assess knowledge, attitude, and perceptions of participants including large demographics factors that influence the knowledge and attitude of the general population [38].

This knowledge-based survey suggest that the people of Pakistan that participated in this study had an average knowledge (50.4%) about the vaccine, its side effects, allergic reactions, and its effect on autoimmune diseases. Knowledge was considerably linked with education, employment status, and residence. This finding is in contrast with the knowledge, attitude, and perception survey conducted in Bangladesh, where knowledge was significantly associated with education, family type, and monthly income of a family [38]. 

The findings of our survey suggest that the mean knowledge score was found to be higher for female participants, in respondents of the age group 21–40, among graduates, and in unmarried participants. These findings concur with two previous surveys conducted in China and the USA. Data from this survey also indicate that gender and education level could have a constructive impact on the knowledge field of participants [39,40]. In our survey, 76.2% of participants had a lack of knowledge regarding the safety of the COVID-19 vaccination in pregnant women. These findings stress the need to convey effective and updated information for the general population through various social media platforms.

Regarding the attitude domain of this study, a mean attitude score is more associated with females than males. This finding is in line with the results of the studies conducted in Indonesia and Bangladesh [41,42]. We believe this result can be of significant value by appealing to women with a domestic level of education and an encouragement for COVID-19 vaccination could strongly suggest the way to a drastic enhancement in the vaccination program. The findings of a high level of a positive attitude of participants towards the preventive measure of vaccination is also reported globally [42].

Our findings show that 64.3% of the participants were willing to take the COVID-19 vaccination without any hesitation, and 75.3% advised their family, friends, and relatives to also take the COVID-19 vaccination. Findings from our study illustrate the wide scale of variation among countries. A study conducted in France during the pandemic shows that 77% of their participants would agree to take the vaccination [43]. While comparing attitude globally in terms of willingness to take the vaccine, studies show that a high percentage of positive attitude responses come from Panama (87.44%), a lower reaction was from Russia (51.34%), Australia had the highest response (92.88%), while the very lowest response was observed in Egypt (43.55%) for taking the COVID-19 vaccination [44].

An average number of participants (51.8%) in our study agreed that the vaccine is safe. This limited knowledge regarding safety of vaccination may be due to rumors and misinformation related to safety issues of the vaccine. Since the pandemic is generally accepted to have started in December 2019, there was only limited knowledge about the disease, along with rumors and misinformation that affected its perception globally [45].

In the perception domain, 57% of participants believe that COVID-19 vaccination had side effects. This apprehension may be due to misinformation regarding fatal and adverse events associated with the COVID-19 vaccine [46,47].

Overall, in our study, female participants had a better ranking of knowledge, attitude, and perception, as compared to males, which is in agreement with findings of prior studies [48]. Possible reasons identified for this were education and socio-economic factors [49,50].

Equal participation by both genders is important for any social survey. This is because both males and females are equally important for forming an opinion about any critical social issues, such as the COVID-19 vaccination. However, in our study, female participation was found to be less than males, which agrees with a previous knowledge, attitude, and perception survey [51]. These findings suggest that more focused research needs to be conducted to determine the possible barriers that women might be facing in participating in such responses. 

In this survey, graduates and postgraduates illustrated high scores towards knowledge, attitude, and perception. This finding suggests that education plays an important role to overcome such pandemics since educated citizens had a greater tendency to analyze the critical situations and consequently behave positively. 

Various research studies have been conducted to initiate effective strategies in order to improve the vaccination rate. These studies show that information alone has a limited impact on enhancing the vaccination rate. Acceptance and a willingness of vaccination is still an unparalleled challenge. Data of this survey could strengthen the efforts of health authorities to achieve their targets of high vaccination coverage through effective communication and updated information.

### Limitations

This survey was conducted over a short time period with incompetence to reach people residing in far-off, remote locations with no access to the Internet, and therefore the findings obtained in this survey might not express the perspective of the whole general population, and the sample was not generalized to a meaningful population. The general population who do not have Internet access and were not proficient in working with online platforms were difficult to connect with. The study used a virtual self-reporting system that may be exposed to social acceptability and memory biases. There would be a response biasness, too, about being judged on knowledge or on financial situations, that also resulted in low responses being one of the drawbacks of online survey.

## 5. Conclusions

Until the development of vaccines, the COVID-19 pandemic was a major global threat. Our survey reflects a poor knowledge (50.6%), fair attitude (75.1%), and poor perception (58.1%) towards vaccination. These findings suggest that more educational campaigns and the advertisement of the correct information status could contribute fairly to eradicating the pandemic. Guiding principle makers can take preliminary steps to ensure the distribution of positive information about the attitudes and perceptions towards COVID-19 vaccinations in order to decrease the vaccine timidity and to increase the vaccination rate. However, research should be performed on participants not included in this study, such as immigrants and the elderly, who do not have access to social media and technology.

## Figures and Tables

**Table 1:** Demographic characteristics of study participants.

Demographics		Frequency (*n*)	Percentages (%)
Age	<20	39	9.7
	21–40	218	54.5
	41–60	109	27.2
	>61	34	8.5
Gender	Female	186	46.5
	Male	214	53.5
Marital status	Unmarried	180	45
	Married	220	55.0
Education level	High school or below	172	43.2
	Graduate	121	30.3
	Postgraduate	107	26.7
Employment status	Gov. employee	81	20.3
	Non Gov. employee	75	18.9
	Self employed	32	8.0
	Student	109	27.3
	Retired	36	9.0
	Unemployed	67	16.9
Residence	Urban	281	70.2
	Rural	119	29.8

Note: number of participants (n); percentages (%) n = 400.

**Table 2:** Categorization of participants and association of demographics with knowledge, attitude and perception.

	Knowledge					Attitude				Perception			
Variables		Good	Fair	Poor	*p* Value	Good	Fair	Poor	*p* Value	Good	Fair	Poor	*p* Value
Age	<20	4	25	7	0.347	20	13	3	**0.001**	8	10	18	0.064
	21–40	43	142	29		132	54	28		39	112	63	
	41–60	26	64	15		35	44	26		22	54	29	
Gender	>61	10	15	5		10	10	10		8	16	5	
	Female	32	129	25		103	56	27	0.148	31	96	59	0.077
	Male	51	129	30		96	74	40		54	100	55	
Marital status	Unmarried	40	115	24	0.831	105	49	24	**0.005**	37	80	62	0.088
	Married	44	144	32		94	82	44		49	116	54	
Education level	high school or below	38	100	34	**0.043**	66	72	33	**0.001**	39	82	50	0.577
	graduate	27	83	11		67	34	20		28	63	30	
	postgraduate	19	75	11		66	24	15		19	50	36	
Employment status	Gov. employee	9	62	10	**0.030**	38	32	11	**0.019**	21	40	20	0.615
	non Gov. employee	13	53	9		42	22	11		13	39	23	
	self employed	9	17	4		7	15	8		10	13	7	
	Student	27	63	17		64	25	17		19	55	33	
	Retired	14	15	7		12	14	10		10	17	8	
	Unemployed	12	47	8		34	22	11		13	30	24	
Residence	Urban	48	187	44	**0.015**	149	85	44	0.051	58	141	80	0.755
	Rural	35	69	13		47	46	24		27	54	35	

Note: for knowledge score 4.8–6 considered (Good) 3.6–4.7 (Fair) and ≤3.5 (Poor); for attitude score 12.8–16 considered Good, 9.6–12.7 Fair and ≤9.5 Poor; for perception score 3.2–4 considered good, 2.4–3.16 Fair and ≤2.3 Poor. *p* value < 0.05 compared within groups. Bold *p* values showed significant association.

**Table 3:** Association within groups with mean knowledge, mean attitude and mean perception score.

	Mean Knowledge		Mean Attitude		Mean Perception	
Variables	Mean (S.D)	*p* Value	Mean (S.D)	*p* Value	Mean (S.D)	*p* Value
Age	0.5602 (0.165) 0.5964 (0.19568) 0.3759 (0.26572) 0.1944 (0.19615)	**0.009**	1.5556 (0.28730) 1.5736 (0.37016) 1.4029 (0.34624) 1.3375 (0.39000)	**0.001**	0.6111 (0.24960) 0.6752 (0.21860) 0.4762 (0.29114) 0.3750 (0.29906)	**0.001**
<20 21–40 41–60 >61						
Gender	0.5658 (0.20926) 0.4525 (0.26746)	**0.001**	1.5390 (0.36552) 1.4705 (0.37266)	**0.066**	0.6452 (0.23052) 0.5226 (0.30705)	**0.017**
Female Male						
Marital Status	0.6134 (0.19448) 0.4188 (0.25468)	**0.012**	1.5475(0.37851) 1.4662(0.36106)	**0.029**	0.6508 (0.23912) 0.5239 (0.29792)	**0.002**
Unmarried Married						
Education Level	0.3777 (0.25744) 0.6074 (0.19467) 0.5997 (0.19107)	**0.001**	1.4523 (0.33256) 1.5289 (0.38587) 1.5560 (0.40541)	0.051	0.4753 (0.29452) 0.6426(0.26581) 0.6810 (0.20655)	**0.001**
High school or below Graduate Postgraduate						
Employment Status	0.5251 (0.20190) 0.5431 (0.23216) 0.2889 (0.25496) 0.6184(0.19018) 0.2269 (0.23622) 0.5075 (0.24521)	**0.001**	1.5231 (0.29981) 1.5724 (0.33364) 1.2750 (0.40921) 1.5339 (0.39624) 1.3819 (0.31756) 1.5037 (0.41342)	**0.002**	0.5494 (0.30726) 0.5967 (0.25959) 0.5250 (0.33701) 0.6752 (0.21504) 0.3750 (0.29580) 0.5821 (0.26610)	**0.001**
Gov. employee Non Gov. employee Self employed Student Retired Unemployed						
Residence	0.5446 (0.21737) 0.4074 (0.29152)	**0.019**	1.5302 (0.35036) 1.4306 (0.41015)	**0.015**	0.6246 (0.26132) 0.4722 (0.29503)	**0.026**
Urban Rural						

Note: S.D is standard deviation. Statistics: one way ANOVA and independent *t* test; *p* value < 0.05 compared within groups. Bold *p* values showed significant association.

**Table 4:** Analysis of factors affecting knowledge, attitude, and perception score.

Independent Variable	Knowledge			Attitude			Perception		
	R Square	0.316		R Square	0.063		R Square		0.224
	Adjusted R Square	0.293		Adjusted R Square	0.031		Adjusted R Square		0.198
	Durbin Watson	1.657		Durbin Watson	1.938		Durbin Watson		1.718
	B	SE	*p*-Value	B	SE	*p*-Value	B	SE	*p*-Value
(Constant)	0.622	0.057	0.000	1.641	0.090	0.000	0.678	0.066	0.000
Age	−0.070	0.028	**0.013**	−0.073	0.045	0.106	−0.069	0.033	**0.036**
Gender	0.007	0.040	0.869	0.024	0.063	0.704	−0.033	0.046	0.471
Marital status	0.076	0.047	0.110	−0.053	0.076	0.483	0.054	0.055	0.329
Education level	0.077	0.025	**0.002**	0.013	0.040	0.751	0.076	0.029	**0.009**
Employment status	−0.028	0.010	**0.006**	−0.015	0.016	0.349	−0.021	0.012	0.067
Residence	−0.075	0.034	**0.030**	−0.034	0.055	0.533	−0.140	0.040	**0.001**

Note: B = unstandardized regression coefficient; SE = Standard error; *p* value < 0.05 considered significant. Bold *p* values showed significant association. Statistics: Multiple linear regression model; *p* value < 0.05 compared within groups.

## References

[1] Lim Y., Ng Y., Tam J., Liu D. (2016). Human Coronaviruses: A Review of Virus–Host Interactions. Diseases.

[2] Abid K., Bari Y.A., Younas M., Tahir Javaid S., Imran A. (2020). Progress of COVID-19 Epidemic in Pakistan. Asia-Pac. J. Public Health.

[3] Kim E.S., Chin B.S., Kang C.K., Kim N.J., Kang Y.M., Choi J.P., Oh D.H., Kim J.-H., Koh B., Kim S.E. (2020). Clinical course and outcomes of patients with severe acute respiratory syndrome coronavirus 2 infection: A preliminary report of the first 28 patients from the korean cohort study on COVID-19. J. Korean Med. Sci..

[4] Lei S., Jiang F., Su W., Chen C., Chen J., Mei W., Zhan L.-Y., Jia Y., Zhang L., Liu D. (2020). Clinical characteristics and outcomes of patients undergoing surgeries during the incubation period of COVID-19 infection. EClinicalMedicine.

[5] Xie J., Tong Z., Guan X., Du B., Qiu H. (2020). Clinical Characteristics of Patients Who Died of Coronavirus Disease 2019 in China. JAMA Netw Open.

[6] Gandhi R.T., Lynch J.B., del Rio C. (2020). Mild or Moderate Covid-19. N. Engl. J. Med..

[7] Fu L., Wang B., Yuan T., Chen X., Ao Y., Fitzpatrick T., Li P., Zhou Y., Lin Y.-F., Duan Q. (2020). Clinical characteristics of coronavirus disease 2019 (COVID-19) in China: A systematic review and meta-analysis. J. Infect..

[8] Helmy Y.A., Fawzy M., Elaswad A., Sobieh A., Kenney S.P., Shehata A.A. (2020). The COVID-19 pandemic: A comprehensive review of taxonomy, genetics, epidemiology, diagnosis, treatment, and control. J. Clin. Med..

[9] Tang D., Tou J., Wang J., Chen Q., Wang W., Huang J., Zhao H., Wei J., Xu Z., Zhao D. (2020). Prevention and control strategies for emergency, limited-term, and elective operations in pediatric surgery during the epidemic period of COVID-19. World J. Pediatr. Surg..

[10] Chan J.F.-W., Yuan S., Kok K.-H., To K.K.-W., Chu H., Yang J., Xing F., Liu J., Yip C.C.-Y., Poon R.W.-S. (2020). A familial cluster of pneumonia associated with the 2019 novel coronavirus indicating person to person transmission: A study of a family cluster. Lancet.

[11] Chirwa G.C. (2020). “Who knows more, and why?” Explaining socioeconomic-related inequality in knowledge about HIV in Malawi. Sci. Afr..

[12] UNICEF (2021). Another 1.2 Million Doses of COVID-19 Vaccine Reach Pakistan through COVAX. https://www.unicef.org/pakistan/press-releases/another-12-million-doses-covid-19-vaccine-reach-pakistan-through-covax.

[13] Farooq U., Pakistan to Receive 13 Million Doses of Pfizer Vaccine—Minister (2021). Reuters. Sec. Asia Pacific..

[14] Hussain S., Pakistan Set to Procure 30 Million Doses of Coronavirus Vaccine (2021). Yahoo! News. https://in.news.yahoo.com/pakistan-set-procure-30-million-120755420.html.

[15] Widakuswara P. (2021). US Ships Moderna Vaccine to Pakistan Amid Delta Variant Surge | Voice of America—English. Voice of America. https://www.voanews.com/covid-19-pandemic/us-ships-moderna-vaccine-pakistan-amid-delta-variant-surge.

[16] Shahzad A., Pakistan Commits $1.1 Bln for COVID Vaccine to Cover Eligible Population (2021). Reuters. Sec. Asia Pacific..

[17] Government of Pakistan (2021). Covid-19 Situation. https://covid.gov.pk/.

[18] Gavi the Vaccine Alliance (2016). Pakistan Progressing on Immunization Efforts. https://www.gavi.org/news/media-room/pakistan-progressing-immunisation-efforts.

[19] Khan M.S., Improving the Covid-19 Vaccination Rate in Pakistan—A Multipronged Policy Approach Front. Public Health 2021..

[20] Reiter P.L., Pennell M.L., Katz M.L. (2020). Acceptability of a COVID-19 vaccine among adults in the United States: How many people would get vaccinated?. Vaccine.

[21] Akbulut S., Gokce A., Boz G., Saritas H., Unsal S., Ozer A., Akbulut M.S., Colak C. (2022). Evaluation of Vaccine Hesitancy and Anxiety Levels among Hospital Cleaning Staff and Caregivers during COVID-19 Pandemic. Vaccines.

[22] İkiışık H., Akif Sezerol M., Taşçı Y., Maral I. (2021). COVID-19 vaccine hesitancy: A community-based research in Turkey. Int. J. Clin. Pract..

[23] Khamis F., Badahdah A., Al Mahyijari N., Al Lawati F., Al Noamani J., Al Salmi I., Al Bahrani M. (2022). Attitudes Towards COVID-19 Vaccine: A Survey of Health Care Workers in Oman. J. Epidemiol. Glob. Health.

[24] Malik A.A., McFadden S.A.M., Elharake J., Omer S.B. (2020). Determinants of COVID-19 vaccine acceptance in the US. EClinicalMedicine.

[25] Yakut S., Karagülle B., Atçalı T., Öztürk Y., Açık M.N., Çetinkaya B. (2021). Knowledge, attitudes, practices and some characteristic features of people recovered from COVID-19 in Turkey. Medicina.

[26] Al-Marshoudi S., Al-Balushi H., Al-Wahaibi A., Al-Khalili S., Al-Maani A., Al-Farsi N., Al-Jahwari A., Al-Habsi Z., Al-Shaibi M., Al-Msharfi M. (2021). Knowledge, attitudes, and practices (Kap) toward the covid-19 vaccine in oman: A pre-campaign cross-sectional study. Vaccines.

[27] Neumann-Böhme S., Varghese N.E., Sabat I., Barros P.P., Brouwer W., van Exel J., Stargardt T. (2020). Once we have it, will we use it? A European survey on willingness to be vaccinated against COVID-19. Eur. J. Health Econ..

[28] Akhu-Zaheya L.M., Jagbir M.T., Othman A., Ahram M. (2014). Media use for seeking health/cancer-related information: Findings from knowledge, attitudes and practices towards cancer prevention and care survey in Jordan. Int. J. Nurs. Pract..

[29] Gallè F., Sabella E.A., Roma P., Da Molin G., Da Molin G., Diella G., Montagna M.T., Ferracuti S., Liguori G., Orsi G.B. (2021). Acceptance of covid-19 vaccination in the elderly: A cross-sectional study in Southern Italy. Vaccines.

[30] Lin Y., Hu Z., Zhao Q., Alias H., Danaee M., Wong L.P. (2020). Understanding COVID-19 vaccine demand and hesitancy: A nationwide online survey in China. PLoS Negl. Trop. Dis..

[31] Palamenghi L., Barello S., Boccia S., Graffigna G. (2020). Mistrust in biomedical research and vaccine hesitancy: The forefront challenge in the battle against COVID-19 in Italy. Eur. J. Epidemiol..

[32] MacDonald N.E., Smith J., Appleton M. (2012). Risk perception, risk management and safety assessment: What can governments do to increase public confidence in their vaccine system?. Biologicals.

[33] Papagiannis D., Malli F., Raptis D.G., Papathanasiou I.V. (2020). Assessment of knowledge, attitudes, and practices towards new coronavirus (SARS-CoV-2) of health care professionals in Greece before the outbreak period. Int. J. Environ. Res. Public Health.

[34] Harkness J.A., Schoua-Glusberg A. (1998). Questionnaires in Translation. ZUMA-Nachr. Spez..

[35] (2008). A Guide To Developing Knowledge, Attitude and Practice Surveys.

[36] Akalu Y., Ayelign B., Molla M.D. (2020). Knowledge, attitude and practice towards covid-19 among chronic disease patients at addis zemen hospital, Northwest Ethiopia. Infect. Drug Resist..

[37] Policy H. (2020). The Knowledge and Attitude of the Community from the Aseer Region, Saudi Arabia, Toward COVID-19 and Their Precautionary Measures Against the Disease. Risk Manag. Healthc. Policy.

[38] Goyal L., Zapata M., Ajmera K., Chaurasia P., Pandit R., Pandit T. (2022). A Hitchhiker’s Guide to Worldwide COVID-19 Vaccinations: A Detailed Review of Monovalent and Bivalent Vaccine Schedules, COVID-19 Vaccine Side Effects, and Effectiveness Against Omicron and Delta Variants. Cureus.

[39] Saiful Islam M., Siddique A.B., Akter R., Tasnim R., Safaet M., Sujan H., Ward P.R., Sikder M.T. (2021). Knowledge, attitudes and perceptions towards COVID-19 vaccinations: A cross-sectional community survey in Bangladesh. BMC Public Health.

[40] Fu C., Wei Z., Pei S., Li S., Sun X., Liu P. (2020). Acceptance and preference for COVID-19 vaccination in health-care workers (HCWs). medRxiv.

[41] Larson H.J., Smith D.M.D., Paterson P., Cumming M., Eckersberger E., Freifeld C.C., Ghinai I., Jarrett C., Paushter L., Brownstein J.S. (2013). Measuring vaccine confidence: Analysis of data obtained by a media surveillance system used to analyse public concerns about vaccines. Lancet Infect. Dis..

[42] Harapan H., Anwar S., Bustaman A., Radiansyah A., Angraini P., Fasli R., Salwiyadi S., Bastian R.A., Oktiviyari A., Akmal I. (2016). Modifiable determinants of attitude towards dengue vaccination among healthy inhabitants of Aceh, Indonesia: Findings from a community-based survey. Asian Pac. J. Trop. Med..

[43] Ferdous M.Z., Islam M.S., Sikder M.T., Mosaddek A.S.M., Zegarra-Valdivia J.A., Gozal D. (2020). Knowledge, attitude, and practice regarding COVID-19 outbreak in Bangladesh: An online-based cross-sectional study. PLoS ONE.

[44] Detoc M., Bruel S., Frappe P., Tardy B., Botelho-Nevers E., Gagneux-Brunon A. (2020). Intention to participate in a COVID-19 vaccine clinical trial and to get vaccinated against COVID-19 in France during the pandemic. Vaccine.

[45] Mannan K.A., Farhana K.M. (2021). Knowledge, Attitude and Acceptance of a COVID-19 Vaccine: A Global Cross-Sectional Study. SSRN Electron. J..

[46] Lazarus J.V., Ratzan S.C., Palayew A., Gostin L.O., Larson H.J., Rabin K., Kimball S., El-Mohandes A. (2021). A global survey of potential acceptance of a COVID-19 vaccine. Nat. Med..

[47] Lombardi A., Bozzi G., Ungaro R., Villa S., Castelli V., Mangioni D., Muscatello A., Gori A., Bandera A. (2021). Mini Review Immunological Consequences of Immunization With COVID-19 mRNA Vaccines: Preliminary Results. Front. Immunol..

[48] Voysey M., Clemens S.A.C., Madhi S.A., Weckx L.Y., Folegatti P.M., Aley P.K., Angus B., Baillie V.L., Barnabas S.L., Bhorat Q.E. (2021). Safety and efficacy of the ChAdOx1 nCoV-19 vaccine (AZD1222) against SARS-CoV-2: An interim analysis of four randomised controlled trials in Brazil, South Africa, and the UK. Lancet.

[49] Al-Zalfawi S.M., Rabbani S.I., Asdaq S.M.B., Alamri A.S., Alsanie W.F., Alhomrani M., Mohzari Y., Alrashed A.A., AlRifdah A.H., Almagrabe T. (2021). Public knowledge, attitude, and perception towards COVID-19 vaccination in Saudi Arabia. Int. J. Environ. Res. Public Health.

[50] Green M.S., Abdullah R., Vered S., Nitzan D. (2021). A study of ethnic, gender and educational differences in attitudes toward COVID-19 vaccines in Israel—Implications for vaccination implementation policies. Isr. J. Health Policy Res..

[51] Jabal K.A., Ben-Amram H., Beiruti K., Batheesh Y., Sussan C., Zarka S., Edelstein M. (2021). Impact of age, ethnicity, sex and prior infection status on immunogenicity following a single dose of the BNT162b2 MRNA COVID-19 vaccine: Real-world evidence from healthcare workers, Israel, December 2020 to January 2021. Eurosurveillance.

